# PAHs in Sediments from Amazon Mangrove and Oyster Farming Areas: Sources, Ecological Risks and Potential Toxicity

**DOI:** 10.1007/s00128-026-04264-5

**Published:** 2026-05-23

**Authors:** Cibelle C. L. Brandão, Thaís R. Sousa, James T. Lee, Júlia A. Ferreira-Griz, Dioniso S. Sampaio, Alexandre M. C. Carmo, Eliete Zanardi-Lamardo, Silvia K. Kawakami

**Affiliations:** 1 Programa de Pós-Graduação Em Oceanografia, Instituto de Geociências, Universidade Federal Do Pará, Rua Augusto Corrêa 1, Campus Guamá, Belém, Pará CEP: 66075-110 Brazil; 2 Laboratório de Pesquisa Em Monitoramento Ambiental Marinho, Instituto de Geociências, Universidade Federal Do Pará, Rua Augusto Corrêa 1, Campus Guamá, Belém, Pará CEP: 66075-110 Brazil; 3 Departamento de Oceanografia, Universidade Federal de Pernambuco, Cidade Universitária, Avenida da Arquitetura S/N, Recife, CEP: 50740-550 Brazil; 4 Instituto de Estudos Costeiros, Universidade Federal Do Pará, Campus de Bragança, Alameda Leandro Ribeiro S/NBragança, Pará CEP: 68600-000 Brazil; 5 Laboratório de Oceanografia Física, Instituto de Geociências, Universidade Federal Do Pará, Rua Augusto Corrêa 1, Campus Guamá, Belém, Pará CEP: 66075-110 Brazil

**Keywords:** Environmental protection area, Sediment toxicity, Coastal management, Equatorial margin, Aquaculture

## Abstract

Sixteen polycyclic aromatic hydrocarbons (PAHs) were examined in mangrove sediments from three Amazonian municipalities, known for port operation, fish market (Curuçá), oyster production (Augusto Corrêa) and tourism (Salinópolis). ∑PAH concentrations (4.55–1303 ng g^−1^ dry sediment; mean: 246 ± 644) indicated low to moderate contamination, in the order: Curuçá ≫ Salinópolis > Augusto Corrêa, with the maxima near natural oyster banks (Curuçá) and lowest in the oyster farming area (Augusto Corrêa). Contamination sources and apportionment were investigated using diagnostic ratios and multivariate analysis, estimating 30% from fossil fuel combustion, 59% from biomass/coal burning and 11% from petrogenic and natural sources. Carcinogenic benzo[a]pyrene-equivalent toxicity was below threshold guidelines, but dibenzo[a,h]anthracene exceeded ecological risks and integrative indices in Curuçá and Salinópolis samples. A potential PAH transfer risk from suspended sediments to oyster may occur. Our results contribute to the local socioeconomic basis with environmental quality dataset and baseline PAHs in mangrove areas.

**Supplementary Information:**

The online version contains supplementary material available at 10.1007/s00128-026-04264-5.

## Introduction

Mangroves form tropical or subtropical ecosystems of ecological and socioeconomic value, providing coastal protection, erosion, water purification, carbon sequestration, and nursery habitat for fisheries (Barbier et al 2011; Otieno et al. 2026). Brazil holds the largest continuous mangrove belt, with more than 70% of its coverage concentrated along the Amazon coast (Lacerda et al. 2002). This region and the adjacent continental shelf constitute a biodiversity hotspot, supporting over 3200 aquatic species and playing a critical role in biodiversity conservation, with 67 species already identified as threatened (de Macedo Klautau et al. 2025). The area also contributes to sustainable development by supporting the livelihoods of traditional coastal communities through small-scale extractive activities such as artisanal fishing, crab harvesting, and oyster farming (Walters et al 2008; Sampaio et al. 2017).

Despite being protected by environmental legislation, Amazon mangroves face increasing anthropogenic pressures that compromise the quality and supply of the goods and services. Port activities and petroleum exploration along the equatorial margin (Araújo et al 2020), expanding tourism (Santos et al. 2023), precarious sanitation conditions (Silva et al. 2020; Pantoja et al. 2024), and changes in land use (Ranieri and Robrini, 2016) represent potential sources of contamination in these ecosystems. Such pressures are concerning in the northeastern coast of Pará State, where mangrove areas host port operations, artisanal fish markets, and oyster production systems of direct socioeconomic relevance to low-income communities.

Mangroves simultaneously promote land-sea connectivity and create favorable conditions for the sorption and burial of hydrophobic contaminants, which can impair ecosystem functioning. Among these, polycyclic aromatic hydrocarbons (PAHs) are of great concern due to their persistence, bioaccumulation, toxic, mutagenic, and carcinogenic potential to humans and aquatic organisms (IARC 2010; Long et al. 2000; Kim et al. 2013). PAHs originate from both natural and anthropogenic processes, including incomplete combustion of petroleum derivatives and biomass, as well as oil spills, biosynthesis and volcanic activities, and are distributed across multiple compartments (Yunker et al 2002; Wang et al. 2017). In mangrove sediments, PAHs can reach ecotoxicological levels, particularly in areas influenced by port and petrochemical activities (Pinheiro et al. 2017; Billah et al. 2022). The affinity of PAHs for organic matter and fine sediment particles further promotes their accumulation and long-term persistence in mangrove substrates (Pinheiro et al. 2017; Zanardi-Lamardo et al. 2019).

The urbanized Amazon coast presents PAH concentrations ranging from low to high impact, with hotspots influenced by petrogenic and pyrogenic inputs and under ecological and potential toxicity risks (Rodrigues et al. 2018; Neves et al. 2018; Kawakami et al. 2023). Surface sediments of pristine Amazon estuary have shown mixed pyrogenic sources (~ 55%) associated with low ecological risks (Pichler et al. 2021). Naturally occurring PAHs, such as naphthalene, in estuarine sediments dating prior the peak of population growth in the Amazon coast, reveals that the contribution of these compounds may be erroneously attributed to anthropogenic sources (Neves et al. 2022), highlighting the need for a better understanding of the origins and distribution of PAH in the Amazon coast. The present study evaluated the concentrations of 16 priority PAHs in mangrove surface sediments from three municipalities on the northeastern coast of Pará State, Brazil, encompassing areas under distinct anthropogenic pressures, some within Marine Extractive Reserves (RESEX). The combination of PAH diagnostic ratios, multivariate analytical tools, integrative ecological risks and carcinogenic toxicity were assessed. By generating baseline contamination data for this understudied Amazon coastal region, the results contribute to environmental quality diagnosis and provide scientific evidence to support the management and conservation of socioeconomically relevant mangrove ecosystems.

## Material and Methods

### Study Area and Sampling

**Fig. 1:**
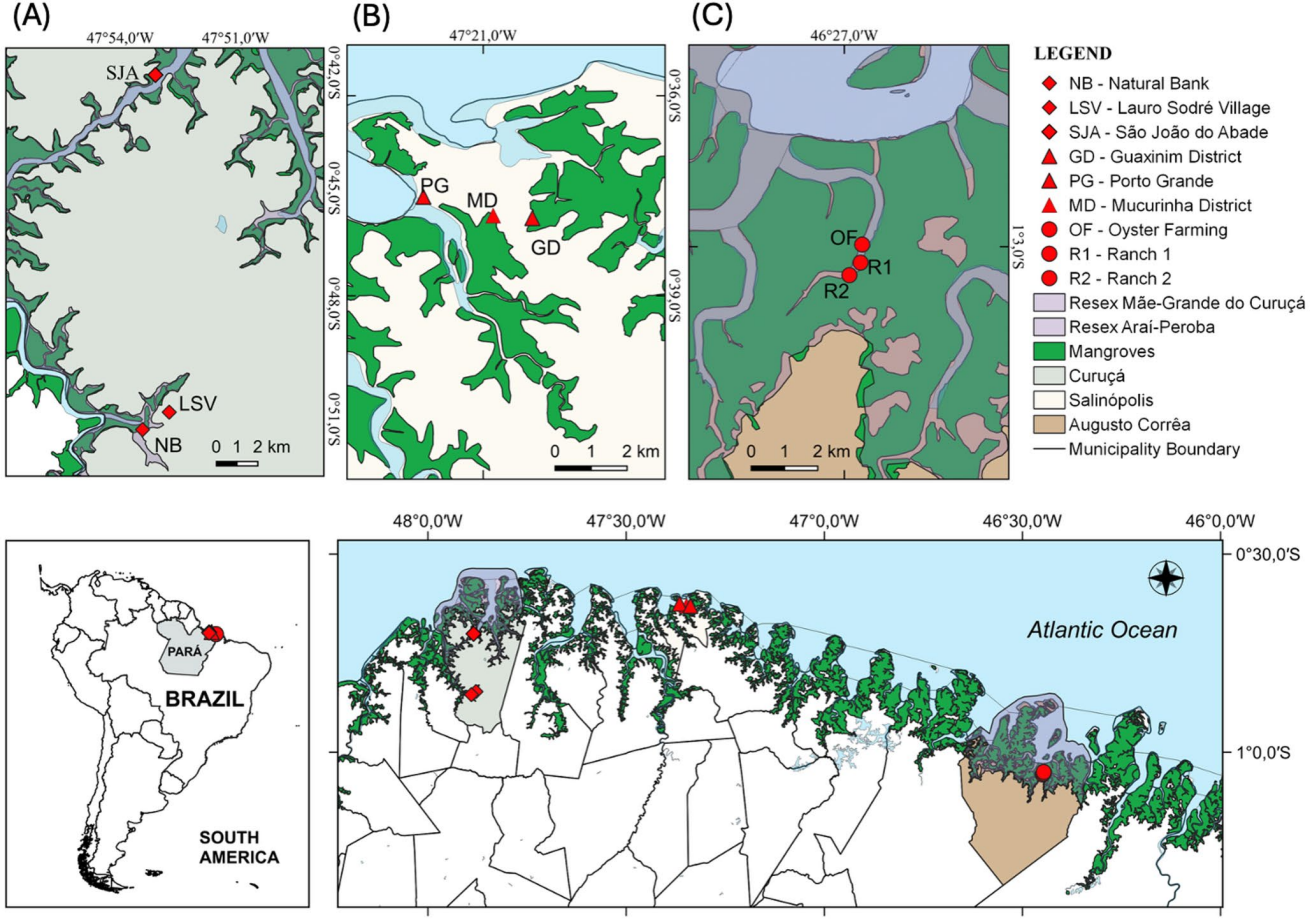
Study area indicating the three municipalities in the Brazilian Amazon coast, Pará State.** A** Curuçá;** B** Salinópolis;** C** Augusto Corrêa

The study area is located on the north coast of Brazil, within the Amazon mangrove belt. The selected sites included areas within and outside Extractive Reserves (RESEX) in three municipalities: Curuçá, Augusto Corrêa and Salinópolis (Fig. 1). A RESEX is a federally designated protected area where traditional communities have the right to sustainably use natural resources for subsistence based on extractivism. Nine sites were chosen for the collection of triplicate surface sediment samples, during low tide, using a 30 cm aluminum core in 2024/2025. When a 30 cm sediment core could not be obtained due to coarser or more fluid substrate, the maximum amount of sediment retrieved was homogenized and used. In Curuçá, sediments were sampled near natural oyster banks, Lauro Sodré Village and São João do Abade Port. In Augusto Corrêa, samples were collected within the RESEX, in the oyster farm area and adjacencies, a remote and preserved mangrove site. In Salinópolis, samples were taken near port and urbanized mangrove areas. Total organic carbon (TOC) and granulometry were measured according to Rodrigues et al. 2018 and Kawakami et al. 2023.

### Analysis of PAHs in Mangrove Sediments

Composite samples were prepared from the mixture of the triplicate sediment samples, and then portioned, freeze-dried, and subjected to extraction and clean up. Approximately 15 g of each sample were placed in extraction cartridges and spiked with 100 µL of a surrogate standard mixture containing deuterated PAHs (acenaphthene-d10, phenanthrene-d10, and chrysene-d12 at 1000 ng mL⁻^1^) to evaluate losses during analytical procedures. Extraction was performed for 8 h in a Soxhlet system using dichloromethane/n-hexane (1:1, v/v), and activated copper pre-treated with 2 M HCl was added to remove sulfur. Extracts were concentrated to 1 mL using a rotary evaporator and purified in adsorption chromatography columns packed with 3.2 g silica and 1.8 g alumina 5% deactivated and preconditioned with n-hexane. Elution was performed with 10 mL of n-hexane to washout aliphatic compounds, and then with 15 mL of dichloromethane/n-hexane (3:7, v/v) to collect PAHs. The eluate was subsequently concentrated to 1 mL. An internal standard mixture (fluorene-d10, benzo[a]anthracene-d12, and benzo[a]pyrene-d12, at 1000 ng mL⁻^1^) was added before instrumental analysis to correct for instrumental variability and determine the surrogate’s recovery. The purified extracts were analyzed in a gas chromatography/mass spectrometry (GC–MS, Agilent 7820A/5975C) equipped with an HP-5MS column (30 m × 0.25 mm × 0.25 µm) in selected ion monitoring (SIM mode) (Arruda-Santos et al. 2018; Zanardi-Lamardo et al. 2019).

### Quality Assurance/Quality Control (QA/QC)

QA/QC included procedural blanks, duplicate samples, matrix spikes and fortified matrices, in each batch analysis. Surrogate recoveries ranged from 42–120% (Table S). The performance of the method was evaluated using NIST 1944 Standard Reference Material, showing a mean recovery of 78 ± 10% and a coefficient of variation of 6.3 ± 4.2%. The limit of quantification (LQ) was established from the lowest concentration of the calibration curve relative to the extracted sediment mass, resulting in 0.067 ng g^−1^ for all analytes. Maps were generated using QGIS version 3.28.13.

### Statistical Analyses and PAH Source Apportionments

Intra-site statistically significant differences among TOC, granulometry and ∑PAH were verified with Kruskal–Wallis test, followed by Dunn's post hoc test. Linear relationships for grain size, TOC, and total and individual PAH concentrations were investigated using Spearman correlation at 0.05 significance level. The sedimentary textural classification was based on Shepard's triangular diagram, using the *ggtern* R package. To investigate the potential contamination sources to the sediments, selected PAH diagnostic ratios were applied (Yunker et al. 2002). A principal component analysis (PCA) was performed using the prcomp function in R Studio (version 4.5.2). ND and < LQ values were replaced by the method detection limit (0.0335 ng g⁻^1^). Data were standardized (z-score) and the analysis was based on the correlation matrix. Source apportionment was assessed using positive matrix factorization (PMF) with the PMF 5.0 Program EPA-USA. Uncertainties were calculated using LQ and relative standard deviations of surrogate recoveries (Cao et al. 2011). Strength categories for ACE, FLU, BaP, IcP, Dah and SumPAH were fixed as weak. A -0.5 Fpeak rotation was applied to the model.

### Sediment Ecological Risks and Toxicity

Sediment ecological risks from PAH were assessed using Sediment Quality Guidelines (SQG) (Long et al 1995; Macdonald et al. 2011) and the Canadian Sediment Quality Criteria (SQC) (Macdonald et al. 1996). Under the SQG framework, potential adverse biological effects of each PAH were interpreted using Effect Range-Low (ERL) and Effect-Range-Median (ERM) thresholds. The Canadian SQC classifies sediments according to individual PAH concentrations, into five effect-probability levels: rare effect level (REL), threshold effect level (TEL), occasional effect level (OEL), probable effect level (PEL), and frequent effect level (FEL). Mean Effects Range Median Quotient (m-ERM-Q) and the Mean Maximum Permissible Concentration Quotient (m-MPC-Q) were included as integrative indices (Table 1S, supplemental material). For m-ERM-Q, risk categories were defined as ≤ 0.1 (low risk, ~ 9% probability of toxicity), 0.11–0.5 (moderate risk, ~ 21%), 0.51–1.5 (high risk, ~ 49%), and > 1.5 (very high risk, > 76%), as applied to mangrove systems (Aghadadashi et al 2019; Yan et al 2023). The m-MPC-Q was used to complement m-ERM-Q by incorporating PAHs lacking ERM values. Potential carcinogenic risk was estimated using toxicity equivalents (TEQ), expressed as BaP-eq/g sediment, as proposed by Nisbet and Lagoy (1992).

**Table 1:**
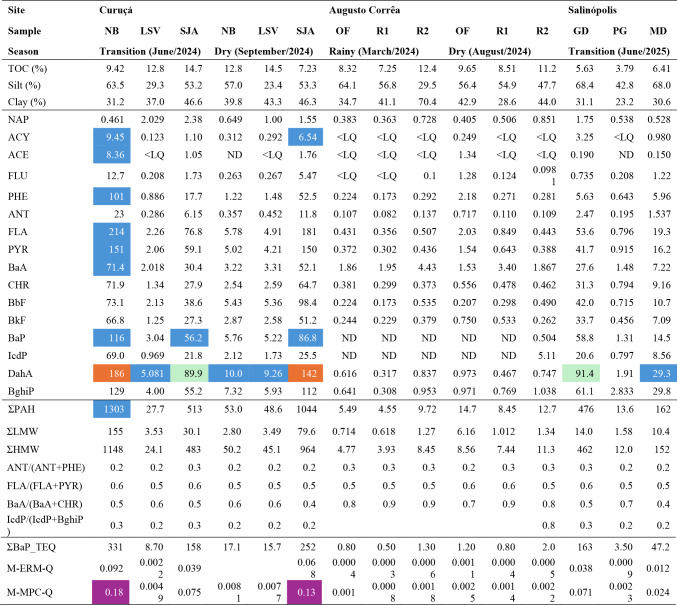
Sedimentary parameters, individual, total and relative PAH concentrations, together with ecological risk and toxic equivalent values. Compound concentrations highlighted in blue, green and orange exceed TEL, ERL or PEL levels, respectively. Values highlighted in purple indicate moderate risk by the integrative index

Legend: TOC: total organic carbon (%); concentrations in ng g^−1^. NB: natural bank; LSV: Lauro Sodré village; SJA: São João do Abade; OF: oyster farm; R1: ranch 1; R2: ranch 2; GD: Guaxinim District; PG: Porto Grande; MD: Mucurinha District. NAP: naphthalene; ACY: acenaphthylene; ACE: acenaphthene; FLU: fluorene; PHE: phenanthrene; ANT: anthracene; FLA: fluoranthene; PYR: pyrene; BaA: benzo[a]anthracene; CHR: chrysene; BbF: benzo[b]fluoranthene; BkF: benzo[k]fluoranthene; BaP: benzo[a]pyrene; IcdP: indeno[1,2,3-cd]pyrene; DahA: dibenzo[a,h]anthracene; BghiP: benzo[g,h,i]perylene. ∑PAH: total concentration of PAHs; ∑LMW: sum of low molecular weight PAHs (2–3 rings); ∑HMW: sum of high molecular weight PAHs (4–6 rings); ∑BaP-TEQ: toxic equivalent quocient based on benzo[a]pyrene (Nisbet and Lagoy 1992); M-ERM-Q: Mean Effects Range Median Quotient; M-MPC-Q: Mean Maximum Permissible Concentration Quotient (Yan et al 2023). ND: not detected; < LQ: below limit of quantification (LQ: 0.067 ng g^−1^)

## Results and Discussion

### PAH Concentrations and Controlling Factors

**Fig. 2:**
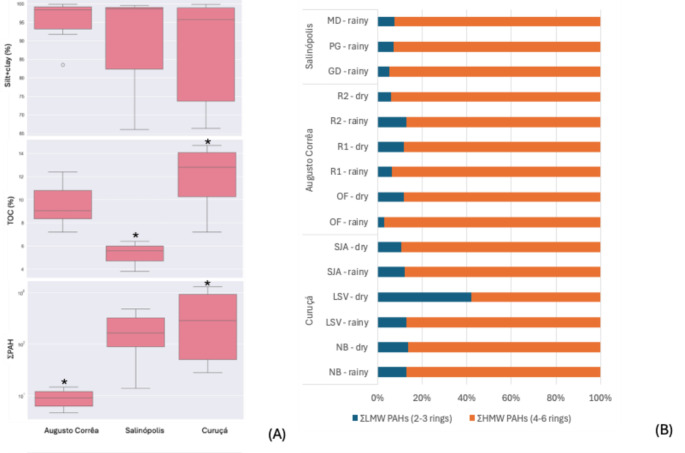
**A** Inter-site comparison of silt + clay (%), TOC (%) and ΣPAH (ng g^−1^). *Significant differences (*p* < 0.05).** B** Percentage of ΣLMW PAHs and ΣHMW PAHs in each sample. NB: natural bank; LSV: Lauro Sodré Village; SJA: São João do Abade; OF: oyster farm; R1: ranch 1; R2: ranch 2; GD: Guaxinim District; PG: Porto Grande; MD: Mucurinha District

Significant differences in ∑PAH concentrations were observed between Curuçá and Augusto Corrêa (*p* < 0.05), but not against Salinópolis (*p* > 0.05, Table 2S). Particle sizes and TOC (%) did not show intra-site differences (*p* > 0.05, Table 2S). No linear correlation was evidenced between silt + clay or TOC with total or individual PAHs (Fig. 1S). In Amazonian settings, the lack of a simple linear relationship for sedimentary data may reflect the occurrence of multiple processes, such as hydrodynamic mixing, episodic deposition, resuspension, and differential degradation under redox conditions (Kawakami et al. 2024). Nevertheless, the inter-site characteristics are best visualized by the combined sediment parameters showing (Fig. 2A):

(i) The magnitude and variability (coefficient of variation, CV) of the PAH load: overall, ΣPAH concentration range followed Curuçá (27.7–1303 ng g^− 1^, CV: 98.8%) > > Salinópolis (13.6–476 ng g^− 1^, CV: 88.7%) > Augusto Corrêa (4.55–14.7 ng g^− 1^, CV: 39.0%) (Table 1). Curuçá hosted the maximum ΣPAH near the natural oyster bank (NB: 1303 ng g^− 1^) and port (SJA: 1044 ng g^− 1^). Lowest PAH concentrations were observed in Augusto Corrêa, a more remote region where oyster production is greatest (OF: 5.49–14.7 ng g^− 1^). The PAH profiles indicated high proportions of PHE, FLA, PYR, BaP, DahA and BghiP (Table 1). The observed ΣPAH concentrations are classified as low to moderate (Baumard et al. 1998). The low PAH concentrations in Augusto Corrêa are similar to previous studies undertaken in remote estuarine areas of the Amazon coast and indicate background levels (Rodrigues et al. 2018; Pichler et al. 2021).
(ii) The pervasive dominance of high molecular weight PAH (HMW, 4–6 rings) over low molecular weight PAH (LMW, 2–3 rings): the HMW PAHs dominated across all municipalities and resulted in ΣLMW/ΣHMW ratio < 1 (Table 1; Fig. 2B). This pattern reflects both combustion inputs and selective preservation processes. HMW PAHs tend to be more hydrophobic, sorb more efficiently to organic matter, and persist longer, whereas LMW compounds are more susceptible to volatilization, dissolution, and biodegradation (Muangchinda et al. 2013).
(iii) TOC abundance and fine particles (silt+clay) typically enhance the accumulation and stabilization of PAHs, especially HMW PAHs, due to greater specific surface area and affinity for organic phases (Yunker et al. 2002; Yan et al. 2023): While all sites exhibited a predominant fine-grained texture and moderate to high TOC content, PAH accumulation varied across the sites. In Curuçá, the highest and most variable HMW PAH concentrations were recorded (ΣHMW: 24.1–1148 ng g^− 1^, CV: 96%) which combined elevated TOC (7.2–14.7%, CV: 16.0%) with consistent fine matrix (silt+clay: 66.3–99.6%, CV: 17.3%). Salinópolis followed a similar granulometric pattern (silt+clay: 66.0-99.5%, CV: 17.7%) but exhibited intermediate ΣHMW levels (ΣHMW: 12.0-461.7%, CV: 90.1%) and lowest TOC content (3.79–6.41%, CV: 20.6%). In contrast, Augusto Corrêa samples featured the highest fine fraction with lowest textural variability (silt+clay: 83.5–99.9%, CV: 6.19%), and highest TOC% (7.25–12.4%, CV: 18.7%), yet it yielded the lowest ΣHMW load (3.94–11.3 ng g^− 1^, CV: 33.4%). The dataset suggests that PAH variability in these mangrove areas is more closely associated with episodic input pulses or spatial heterogeneity in source proximity than with differences in retention capacity and stabilization.

### PAH sources Inferred from Diagnostic Ratios and Multivariate Analysis

Cross-plots of the PAH diagnostic ratios revealed predominantly pyrogenic origins for the contamination (Fig. 2B). The IcdP/(IcdP + BghiP) vs. FLA/(FLA + PYR) indicates sources related to liquid fuel combustion, suggesting contributions from diesel and gasoline. In the local context, such signature is plausibly linked to boats and other logistic vehicles, whose activities tend to be intense due to ports, villages and fish markets in Curuçá (SJA, LSV), and in addition to tourism in Salinópolis (PG, MD). Biomass burning, including vegetation, wood, coal, and garbage, is also signaled for the three municipalities. The BaA/(BaA + CHR) vs. FLA/(FLA + PYR) corroborates the convergence of multiple ratio frameworks as mixed pyrogenic sources for most sites. The LMW/HMW < 0.2 associated with FLA/(FLA + PYR) between 0.4 and 0.6 highlighted the combustion of petroleum and derivatives in Salinópolis (PG), and HMW PAHs predominance.

The combined use of the diagnostic ratios improves source attribution in Amazon systems, which are prone to intense weathering. More labile isomers may undergo differential alteration during transport, deposition, and diagenesis, which includes photoxidation and transformations within redox microenvironments, shifting diagnostic ratios without necessarily representing a true change in source (Tobiszewski and Namieśnik 2012; Chidewe et al. 2026). Furthermore, endogenous bacteria in the mangrove sediment can degrade LMW PAHs with high efficacy and, thus, affecting ACE, PHE and PYR concentrations (Muangchinda et al. 2013; Li et al. 2022) and diagnostic ratios. High temperatures, stable low pH, reducing characteristics, low salinity and high nutrient contents, typical of Amazon aquatic environments (Kawakami et al. 2023), can either chemically interfere in the preservation of hydrocarbons or enhance metabolism and degradation rates (Li et al. 2024; Mekonnen et al. 2024), and possibly lead to misinterpretation of the signatures. Multivariate analysis helps to overcome some of these problems and to deepen the understanding of the PAH sources (Cao et al. 2011).

**Fig. 3:**
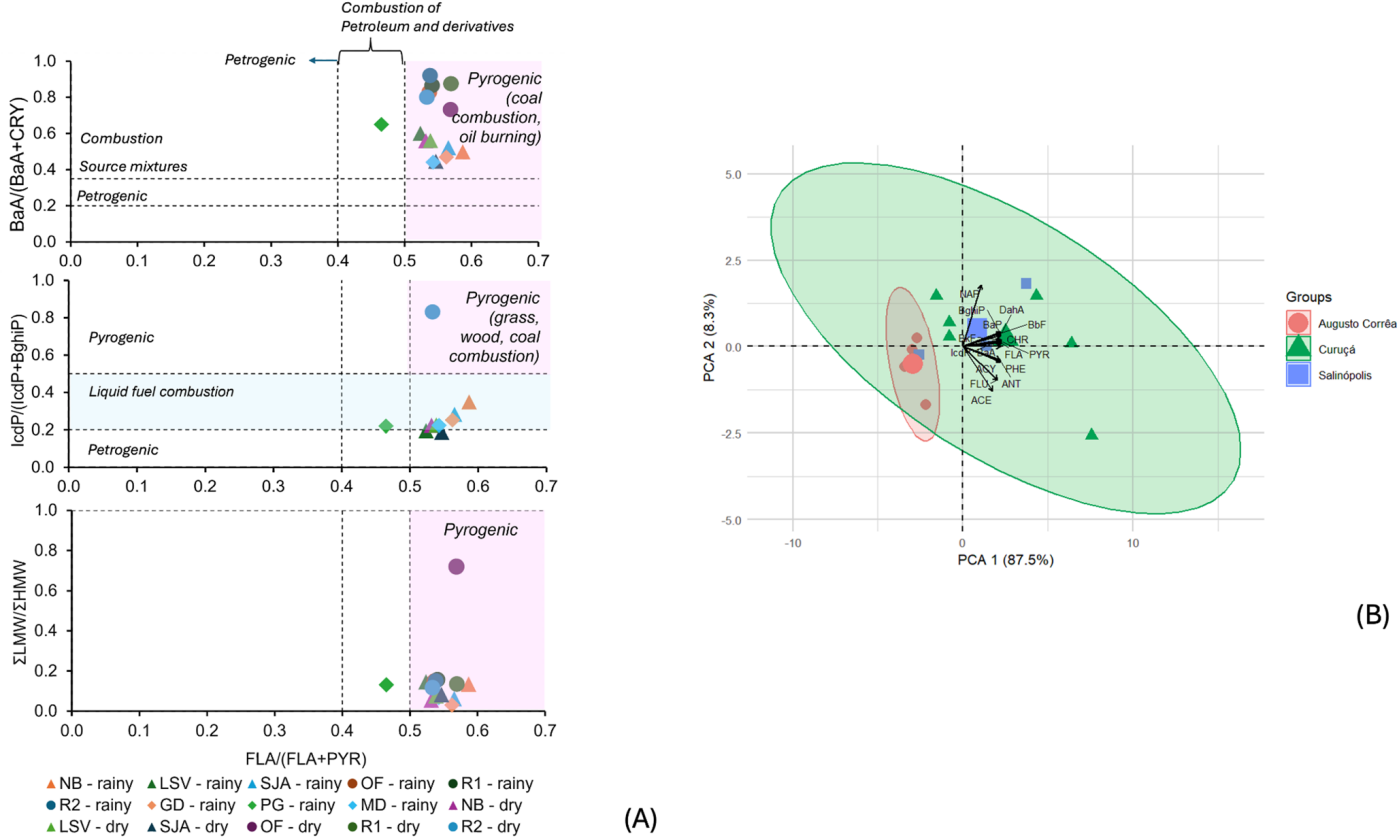
**A** Cross-plots of PAH diagnostic ratios for Amazon mangrove samples: IcdP/(IcdP + BghiP) = indeno[1,2,3-c,d]pyrene/(indeno[1,2,3-c,d]pyrene + benzo[g,h,i]perylene); BaA/BaA + CRY) = benzo[a]anthracene/(benzo[a]anthracene + chrysene); ΣLMW/ΣHMW = ΣLow Molecular Weight/Σ High Molecular Weight PAH. (B) PCA biplot shows individual PAHs and sample locations.** B** PMF model estimates contamination source apportionment

**Fig. 4:**
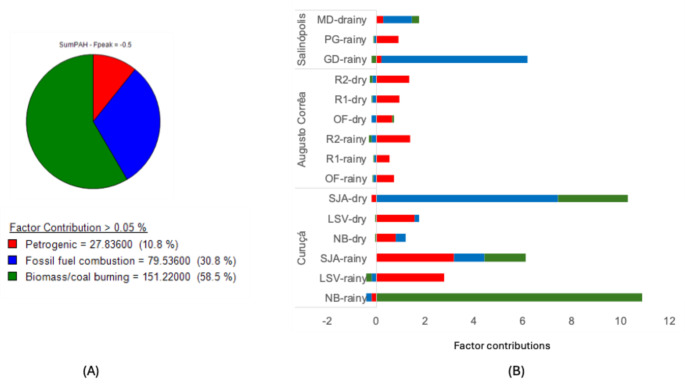
**A** Factor contributions of PAH sources for each sample;** B** Source apportionment for the ∑PAH were identified as Factor 1: petrogenic (unburned petroleum mixed with natural PAHs), Factor 2: fossil fuel combustion, and Factor 3: biomass/coal burning

The PCA explained 87.5% of the total variance by high loadings of 4- to 6-ring PAHs (Fig. 3B), a pyrolytic chemical signature consistent with the diagnostic ratios. While Augusto Corrêa presented clustered and homogeneous samples (red circles) associated with LMW PAHs at lower concentrations, Curuçá stood out for its wide dispersion (green ellipse) and higher concentrations. Salinópolis samples in the center of the biplot reflected an intermediate contamination profile. Overall, the PMF model showed source apportionment of 30% for fossil fuel combustion, 59% for biomass/coal burning and 11% for petrogenic + natural sources (Fig. 4A). Given our sample size (n = 15), it was not possible to discriminate other sources as in similar studies (Cao et al. 2011; Pichler et al. 2021), without risks of overfitting and numerical instability. The model revealed petrogenic plus natural PAH sources for all sites, an undisclosed contribution from the diagnostic ratios (Fig. 4B). For Augusto Corrêa profile, the absence of petrogenic sources suggests a baseline contribution of naturally occurring PAHs, as observed in sediment cores (Neves et al. 2022).

### Ecotoxicological Risks and Toxicity Equivalency

Adverse biological effects investigated through m-ERM-Q index < 0.1 revealed low risk for all sites, whereas m-MPC-Q index ~ 0.11 indicated moderate risk for NB and SJA samples from Curuçá only (Table 1). These findings are comparable to those from low impacted mangrove sites (Yan et al. 2023) and lower than those under petrochemical and port influences (Jafarabadi et al. 2024). In terms of carcinogenic toxicity, TEQ values showed variation between municipalities, with higher values at Curuçá and Salinópolis, NB (331 ng g⁻^1^), SJA (158 ng g⁻^1^) and GD (164 ng g⁻^1^), while Augusto Corrêa maintained values < 1 ng g⁻^1^ at all points (Table 1), indicating more preserved conditions. In comparison with national and international sedimentary guidelines, compounds such as dibenzo[a,h]anthracene (DahA) exceeded reference values at specific points, especially in Curuçá, indicating greater environmental risk. In the Amazon region, the drainage of sewage and dump sites also represent a load of these compounds to the aquatic environment (Sousa et al. 2020). TEQ values that were similar to or more than three times higher than those found in our study were observed in regions hosting the major ports in the SE Brazilian coast (Pinheiro et al. 2017).

To the best of our knowledge, our results are the first to report PAH concentrations and carcinogenic toxicity of Amazonian mangrove sediments in areas of oyster production. Sedimentary PAH concentrations around 800–900 ng g^−1^ are favorable for promoting transfer to oysters, especially to lipid-rich organs (Wang et al. 2020). In general, the high TOC% in mangrove sediments reduces HMW PAH bioavailability, with the soluble LMW PAHs being the preferred fraction for bioaccumulation, as observed for different oyster species (Gan et al. 2021). PAH internal body burden of 300 ng g^−1^ for oyster larvae is harmful for their development (Geffard et al. 2003). Since oysters depend on filter-feeding and ingesting suspended particles, sediment resuspension through bioturbation or cultivation practices can modulate risks, facilitating the uptake of particulate PAHs by oysters. In this situation, the oysters from the natural bank in Curuçá (NB, ∑PAH = 1303 ng g^−1^) are more susceptible to PAH accumulation.

## Conclusion

PAHs exhibited a heterogeneous distribution in mangrove sediments across the three Amazonian municipalities investigated, with a contamination range following Curuçá >  > Salinópolis > Augusto Corrêa. The integrative indices revealed low to moderate risks of the sediment samples, but PAH toxicity equivalency was higher at specific points in Curuçá and Salinópolis. Samples from Augusto Corrêa showed a well-preserved environment, with PAH concentrations being related to a natural background for Amazonian mangrove sediments. Burning of biomass, including coal, wood, garbage, and fossil fuels were identified as the main PAH sources. Currently, sedimentary PAH levels in the oyster farming area of Augusto Corrêa do not appear to pose a direct threat of PAH transfer to oysters, but natural oyster banks in Curuçá are under potential risk by suspended contaminated sediments. Our study indicates the need to safeguard these ecosystems from anthropic pressures, as toxic PAHs were detected. Our data contribute to the local socioeconomic basis with an environmental quality dataset.

## Supplementary Information

Below is the link to the electronic supplementary material.

Supplementary file1 (DOCX 4151 kb)
