# Supplementary material for: PAHs in Sediments from Amazon Mangrove and Oyster Farming Areas: Sources, Ecological Risks and Potential Toxicity
Source: Bull Environ Contam Toxicol. 2026 May 23;116(6):109. doi: 10.1007/s00128-026-04264-5 (PMC13198471; doi:10.1007/s00128-026-04264-5)

**PAHs in sediments from Amazon mangrove and oyster farming areas: sources, ecological risks and potential toxicity**

Cibelle C. L. Brandão^1^; Thaís R. Sousa^1^; Júlia Griz^2^; James T. Lee^3^; Dioniso S. Sampaio^1^; Alexandre M. C. Carmo^4^; Eliete Zanardi-Lamardo^2^; Silvia K. Kawakami^1^*

Corresponding author: [skawakami@ufpa.br](mailto:skawakami@ufpa.br)

Table S. Surrogate recoveries in percentage.

|  | NB-R | LSV-R | SJA-R | NB-D | LSV-D | SJA-D | OF-R | R1-R | R2-R | OF-D | R1-D | R2-D | GD-R | PG-R | MD-R |
| --- | --- | --- | --- | --- | --- | --- | --- | --- | --- | --- | --- | --- | --- | --- | --- |
| Acenafteno-D10 (PI) | 55 | 89 | 88 | 47 | 81 | 65 | 71 | 84 | 71 | 43 | 82 | 73 | 82 | 52 | 58 |
| Fenantreno-D10 (PI) | 108 | 113 | 118 | 93 | 107 | 104 | 119 | 102 | 95 | 64 | 111 | 99 | 120 | 81 | 115 |
| Criseno-D12 (PI) | 42 | 108 | 77 | 89 | 86 | 114 | 72 | 51 | 55 | 50 | 65 | 57 | 119 | 93 | 85 |

Table 1S. Indices for risk assessment of PAH in sediments.

| Indices | Equations |  |
| --- | --- | --- |
| Mean Effects Range Median Quotient (m-ERM-Q) | $m-ERM-Q= \frac{\sum_{i=1}^{n} \frac{Ci}{ERMi}}{n}$ | (1) |
| Mean Maximum Permissible Concentration Quotient (m-MPC-Q) | $m-MPC-Q= \frac{\sum_{i=1}^{n} \frac{Ci}{MPCi}}{n}$ | (2) |
| Toxicity equivalent quocient (TEQ) | $TEQ=\sum_{i=1}^{n} C_{i} TEF_{i}$ | (3) |

*Ci*: individual concentration of the PAH in the sample; *ERMi*: Effects Range Median (ERM) value for the individual PAH; *n*: total number of PAHs with available ERM or MPC values; $\sum\frac{Ci}{MPCi}$ : sum of the quotients of the concentration of each PAH (*Ci*) by its reference value MPC (*MPCi*); *TEFi*: toxic equivalence factor of each PAH in the sample (USEPA 1993). TEQ values: toxicity-weighted masses of PAH mixtures, expressed in terms of benzo(a)pyrene equivalents per gram of sediment.

Table 2S. Statistical comparison between the variables of the three regions.

| Parameter | Kruskal-Wallis | Dun test | Result |
| --- | --- | --- | --- |
| TOC (%) | p = 0.0174 |  | Curuçá ≠ Salinópolis |
| Silt (%) | p = 0.3633 |  | — |
| Clay (%) | p = 0.0672 |  | — |
| ΣPAH |  | p = 0.0077 | Augusto Corrêa ≠ Curuçá |
| ΣPAH |  | p = 0.115 | Augusto Corrêa = Salinópolis |
| ΣPAH |  | p = 0.562 | Curuçá = Salinópolis |

Figure 1S. Spearman correlation matrix (ρ) between PAHs, TOC, and grain size (silt + clay) in mangrove sediments of the Amazon estuary. The grayscale bar represents the correlation strength, ranging from -1 to 1. Numerical values within the squares indicate the correlation coefficient, and red * denote statistical significance at the *p* < 0.05.


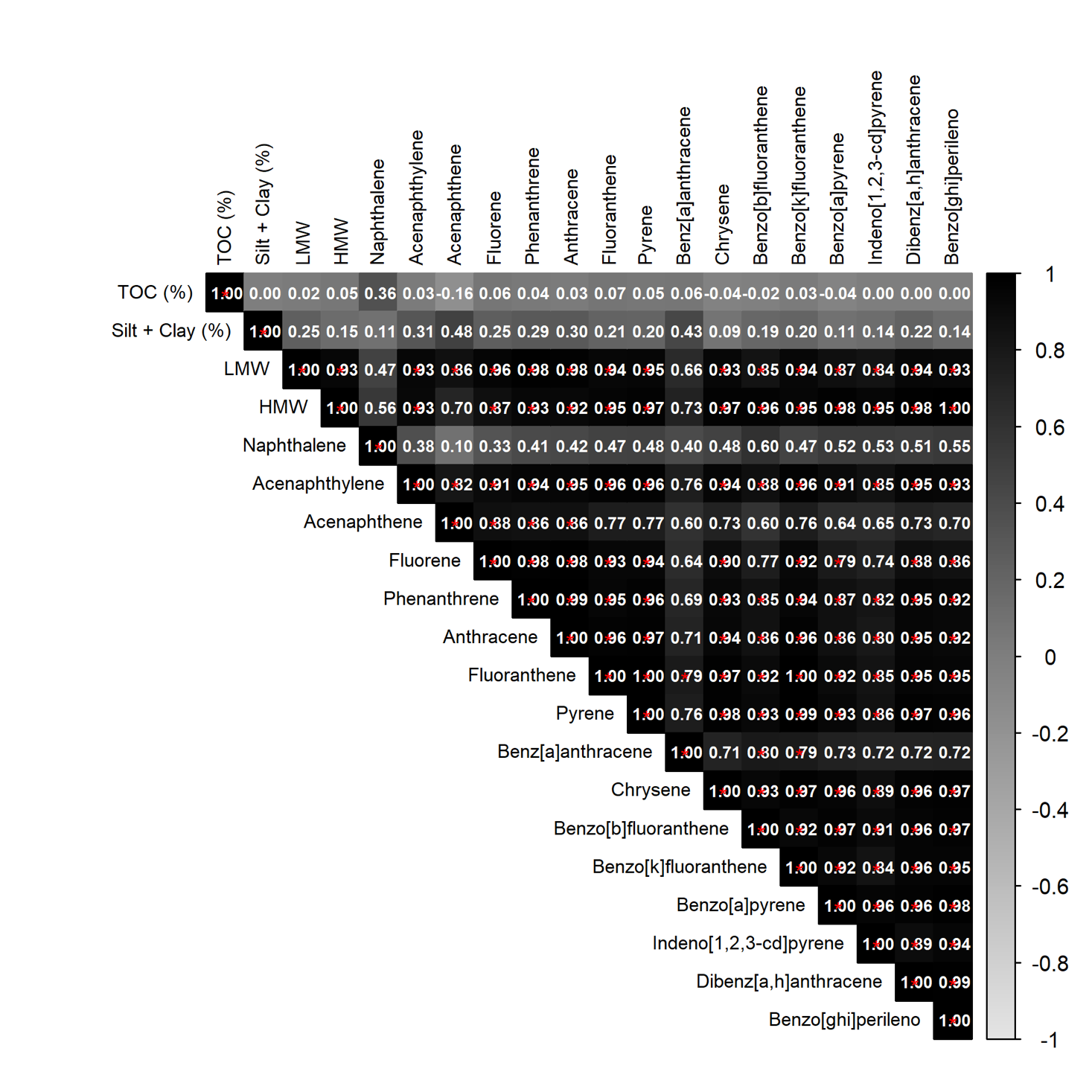

Supplement: Supplementary file 1 — Supplementary file1 (DOCX 4151 kb) [file 128_2026_4264_MOESM1_ESM.docx]
